# Comments on: When technology advances faster than competence: the risk of deskilling in obstetrics and gynecology

**DOI:** 10.61622/rbgo/2026rbgo108L22

**Published:** 2026-10-13

**Authors:** Shigeki Matsubara

**Affiliations:** 1 Jichi Medical University Department of Obstetrics and Gynecology Tochigi Japan Department of Obstetrics and Gynecology, Jichi Medical University, Tochigi, Japan

Dear Editor,

I read with great interest the article by da Silva et al. discussing the risk of deskilling in obstetrics and gynecology (OBGYN) associated with the increasing use of artificial intelligence (AI).^(1)^ The authors appropriately emphasize that excessive reliance on AI may weaken independent clinical reasoning and procedural competence. I agree with this concern. I believe several additional considerations deserve attention.

First, although AI may deskill physicians, discussing future medical practice assuming the absence of AI is no longer realistic. AI is rapidly becoming integrated into many aspects of health care and will likely become part of routine OBGYN practice. Rather than asking whether AI should be used, the more practical question is how physicians can use AI while maintaining the clinical judgment and fundamental skills required for safe patient care.

Second, the authors repeatedly emphasize the danger of deskilled physicians, particularly in emergency situations. I agree that emergencies deserve special attention because AI may not always be available or applicable. The minimum competencies required to recognize and manage common obstetric emergencies should remain mandatory irrespective of technological advances. For example, persistent fetal bradycardia or recurrent late decelerations on cardiotocography require immediate intervention even when AI-assisted interpretation is unavailable. Likewise, massive hemorrhage during placenta percreta surgery requires immediate hemostasis, which cannot be delegated to an algorithm. Such fundamental skills and competencies should remain mandatory, while AI itself may serve as a valuable educational tool to prepare physicians for these situations.

When I was an OBGYN resident 47 years ago, cervical cancer staging depended largely on bimanual and rectal examination to assess parametrial invasion. After I became confident in these skills, ultrasonography, computed tomography, and magnetic resonance imaging became widely available and substantially improved diagnostic accuracy. Although I still value the traditional examination skills I learned, younger generations appropriately use newer technologies to complement their fundamental skills. Similar transitions have occurred repeatedly throughout the history of OBGYN.

The challenge, therefore, is not technological progress itself but achieving an appropriate balance. All physicians should learn to use AI appropriately. Ideally, younger physicians should also acquire the same level of fundamental skills and competence as previous generations. If this ideal cannot always be achieved, educational programs should ensure that physicians preserve the core clinical skills, knowledge, and decision-making abilities necessary for safe practice while developing the skills needed to use AI wisely.

We have already entered the AI era and passed the point of no return. The challenge is therefore not choosing between traditional clinical skills and AI, but integrating them appropriately. Maintaining awareness of the potential for deskilling remains important, but technological innovation and preservation of fundamental clinical skills and competence should be viewed as mutually reinforcing rather than opposing goals.

## Data Availability

The authors did not make the data from this article available in repositories prior to submission.
